# Cancer Stem Cells and Cell Cycle Genes as Independent Predictors of Relapse in Non-small Cell Lung Cancer: Secondary Analysis of a Prospective Study

**DOI:** 10.1093/stcltm/szac040

**Published:** 2022-06-08

**Authors:** Valentina Masciale, Federico Banchelli, Giulia Grisendi, Roberto D’Amico, Antonino Maiorana, Alessandro Stefani, Uliano Morandi, Franco Stella, Massimo Dominici, Beatrice Aramini

**Affiliations:** Division of Thoracic Surgery, Department of Medical and Surgical Sciences, University of Modena and Reggio Emilia, Modena, Italy; Division of Oncology, Department of Medical and Surgical Sciences, University of Modena and Reggio Emilia, Modena, Italy; Center of Medical Statistics, Department of Medical and Surgical Sciences, University of Modena and Reggio Emilia, Modena, Italy; Division of Oncology, Department of Medical and Surgical Sciences, University of Modena and Reggio Emilia, Modena, Italy; Center of Medical Statistics, Department of Medical and Surgical Sciences, University of Modena and Reggio Emilia, Modena, Italy; Institute of Pathology, Department of Medical and Surgical Sciences, University of Modena and Reggio Emilia, Modena, Italy; Division of Thoracic Surgery, Department of Medical and Surgical Sciences, University of Modena and Reggio Emilia, Modena, Italy; Division of Thoracic Surgery, Department of Medical and Surgical Sciences, University of Modena and Reggio Emilia, Modena, Italy; Division of Thoracic Surgery, Department of Experimental, Diagnostic and Specialty Medicine—DIMES of the Alma Mater Studiorum, University of Bologna, G.B. Morgagni—L. Pierantoni Hospital, Forlì, Italy; Division of Oncology, Department of Medical and Surgical Sciences, University of Modena and Reggio Emilia, Modena, Italy; Division of Thoracic Surgery, Department of Medical and Surgical Sciences, University of Modena and Reggio Emilia, Modena, Italy; Division of Thoracic Surgery, Department of Experimental, Diagnostic and Specialty Medicine—DIMES of the Alma Mater Studiorum, University of Bologna, G.B. Morgagni—L. Pierantoni Hospital, Forlì, Italy

**Keywords:** cancer stem cells, cell cycle progression score, cell cycle progression, recurrence, non-small cell lung cancer

## Abstract

**Purpose:**

Cancer stem cells (CSCs) are described as resistant to chemotherapy and radiotherapy. It has been shown that CSCs influence disease-free survival in patients undergoing surgery for lung cancer (NCT04634630). We recently described an overexpression of CSCs recurrence-related genes (RG) in lung cancer. This study aims to investigate CSC frequency and RG expression as predictors of disease-free survival in lung cancer.

**Experimental Design:**

This secondary analysis of a prospective cohort study involved 22 surgical tumor specimens from 22 patients harboring early (I-II) and locally advanced (IIIA) stages ACL and SCCL. Cell population frequency analysis of ALDH^high^ (CSCs) and ALDH^low^ (cancer cells) was performed on each tumor specimen. In addition, RG expression was assessed for 31 target genes separately in ALDH^high^ and ALDH^low^ populations. CSCs frequency and RG expression were assessed as predictors of disease-free survival by Cox analysis.

**Results:**

CSCs frequency and RG expression were independent predictors of disease-free survival. CSC frequency was not related to disease-free survival in early-stage patients (HR = 0.84, 95%CI = 0.53-1.33, *P* = .454), whereas it was a risk factor for locally advanced-stage patients (HR = 1.22, 95%CI = 1.09-1.35, *P* = .000). RG expression—if measured in CSCs—was related to a higher risk of recurrence (HR = 1.19, 95%CI = 1.03-1.39, *P* = .021). The effect of RG expression measured in cancer cells on disease-free survival was lower and was not statistically significant (HR = 1.12, 95%CI = 0.94-1.33, *P* = .196).

**Conclusions:**

CSCs frequency and RG expression are independent predictors of relapse in lung cancer. Considering these results, CSCs and RG may be considered for both target therapy and prognosis.

## Graphical Abstract

**Graphical Abstract:**
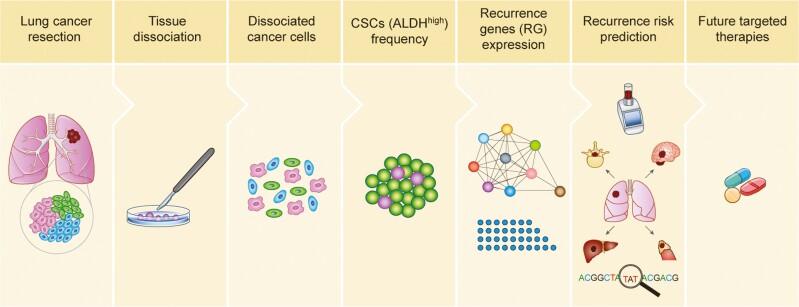

**Lessons Learned**

- Cancer stem cells (CSCs) are described as resistant to chemotherapy and radiotherapy.
- CSCs influence disease-free survival in patients undergoing surgery for lung cancer.
- CSCs have been assessed for the overexpression of a recurrence-related genes (RG) panel.
- CSC frequency and RG expression are independent predictors of relapse in lung cancer, and they may be considered for both target therapy and prognosis.

**Significance Statement**

Cancer stem cell frequency and recurrence-related gene expression are independent predictors of relapse.

## Introduction

Cancer stem cells (CSCs) are tumor-initiating cells that are resistant to conventional cancer therapies, such as chemotherapy and radiotherapy.^1,2^ CSCs are increasingly being described as responsible for tumor recurrence and distant metastasis, leading to treatment failure and poor clinical outcomes in patients with cancer.^3,4^ In vitro studies have shown that CSCs are surprisingly resilient, even in restrictive culture conditions, and highly resistant to cellular stress, allowing them to undergo anchorage-independent growth and survive without sera supplements.^1,2,5^ These experimental findings highlight the potential dangers of CSCs in terms of resistance to common oncological treatments and as inductors of tumor development and progression.^6-8^ Therefore, innovative approaches are needed to address the potential consequences of the presence of CSCs. Recently, in a prospective cohort study, our research group demonstrated the influence of CSCs on disease-free survival in patients undergoing surgery for adenocarcinoma of the lung (ACL) and squamous cell carcinoma of the lung (SCCL).^9^ Although a correlation between recurrence risk and CSCs in early stages has not been found, we observed a positive association between CSC frequency and the risk of relapse in locally advanced-stage patients.^9^ These results highlight the importance of further molecular investigations of the prognostic role of CSCs at different lung cancer stages for achieving a better definition of lung cancer development and progression.^9^

Metastasis, the spreading of cancer cells from a primary tumor site to other tissue and distant organs, is responsible for more than 90% of cancer-related deaths.^10,11^ This is especially true for lung cancer, which shows a postoperative recurrence of 20%-75% during the first 5 years.^7,8,12,13^ Metastasis is the final step in cancer when a cell clone prevails over others because it has the biological characteristics to develop and favor tumor dissemination.^14^ The clone is a CSC that drives tumor development due to its self-renewal ability, uncontrolled proliferation, and genomic instability.^15^ All cancer cells with the capacity to colonize distant organs have the features of CSCs and exert their tumor-initiating capacities under adverse environmental conditions.^16^ The idea that metastasis can be boosted by selected subpopulations of CSCs has emerged over the last 5 years.^17,18^ Currently, consolidated risk stratification models to predict recurrence in this population do not consider genetic and molecular characteristics, likely due to the difficulties related to assessing cancer multi-factors.^19,20^ Bueno et al validated a prognostic score in patients who underwent surgery for early stages of lung adenocarcinoma (ACL) to predict lung cancer mortality.^21^ This score, the cell cycle progression (CCP) score, is a molecular expression signature of 31 cell cycle proliferation genes that identifies early-stage (I-II) patients with a higher risk of cancer-related death after surgical resection in lung adenocarcinoma. We recently described an overexpression of these recurrence-related genes (RG) in CSCs in early and locally advanced stages (IIIA) of ACL and SCCL.^22^ Although these results still need to be replicated in larger cohorts of patients, it could be important to consider these genes for future targeted, stage-tailored therapies, and for risk stratification models.^22^

In our previous research, we have described a positive correlation between CSC frequency and risk of relapse in locally advanced-stage patients, as on average the hazard increased by about 26% for every 1% increase in CSC frequency.^9^ This finding indicates that CSC frequency could represent a strong predictor variable for patient prognosis. Although the average percentage of CSCs was low in both early and locally advanced stages (about 3%), their impact on disease-free survival needs to be further investigated by considering the presence of overexpressed cell cycle genes linked to recurrence.^22^

In light of these considerations and of previously published research,^4,9,21,22^ in the present study, we aim to investigate CSC frequency and RG expression as independent predictors of relapse in lung cancer. A better description of the role of CSCs and RG as predictors of cancer relapse will contribute to the knowledge on these subpopulation of cells as both a therapeutic target and a possible prognostic factor.

## Methods

This study involved the collection of 22 surgical tumor specimens from 22 patients in early (I-II) and locally advanced (IIIA) stages of non-small cell lung cancer (NSCLC). Cell population frequency analysis of ALDH^high^ (CSC) and ALDH^low^ (cancer cells) was performed on each tumor specimen.^5,9^ In addition, RG expression was assessed for 31 target genes (previously validated on adenocarcinoma putative,^21^ separately in ALDH^high^ and ALDH^low^ populations.^22^

### Study Aim and Design

This was a secondary analysis of a prospective cohort study.^9^ The aim was to assess the joint effect of CSC frequency and RG expression on disease-free survival. The study was carried out according to STROBE guidelines.^23^

### Study Population

Patients included in this study were harboring stage I, II, or IIIA (TNM [Tumor, Node, Metastasis], 8th edition) NSCLC,^24^ aged 18-85 years, and undergoing major lung resection by lateral thoracotomy at the Division of Thoracic Surgery of the University Hospital of Modena (Italy) between October 2017 and September 2019. The inclusion criteria were: age between 18 and 85 years; R0 resection, the availability of adequate, fresh surgical specimens preceded by histological examination for diagnosis. Exclusion criteria were incomplete resection; unknown tumor, lymph node, and metastatic status; synchronous tumors; and previous lung cancer.

### Calculation of CSC Frequency

Primary tumor cells harvested from fresh surgical biopsy were stained with ALDEFLUOR Assay (STEMCELL Technologies, Vancouver, BC, Canada) to calculate the frequency of CSCs. Isolation was performed by fluorescence-activated cell sorting (FACS) using a BD FACSAria III (Becton Dickinson, Franklin Lakes, NJ, USA). Cell morphology was assessed using size scattering and forward scattering. Gating strategy included the ALDH^high^ gate, which was set at least one log apart from the ALDH^low^ gate. Results were analyzed using FACS Diva software (Becton Dickinson, Franklin Lakes, NJ, USA), and sorted cells were readily lysed for further gene expression analysis. CSC frequency was calculated as the percentage of ALDH^high^ cells among all viable cells.

### Gene Expression Analysis

RNA was isolated from ALDH^high^ and ALDH^low^ cells using the RNeasy Mini kit (Qiagen) according to the manufacturer’s instructions. Reverse transcription was then performed on 500 ng of total RNA using the RevertAid First Strand cDNA synthesis kit (Thermo Fisher Scientific, Waltham, MA, USA).^21,22^ The resulting cDNA was pre-amplified, diluted in Tris-EDTA (TE) buffer, loaded in TaqMan low-density cards (TLDA; Thermo Fisher Scientific), and run on a QuantStudio 12K Flex Real-Time PCR system to analyze gene expression. TaqMan Universal PCR Master Mix (Thermo Fisher Scientific) was used. Expression data were recorded in duplicates as the cycle threshold (Ct) value—the PCR cycle in which the fluorescence intensity exceeded a predefined threshold—separately in ALDH^high^ (CSC) and ALDH^low^ (cancer cells) populations. The gene panel used for analysis contained 31 RGs^21,22^ and three housekeeping genes: RPL13A, RPL4, and RPS29. Data management of undetermined Ct values has already been described.^22^ The RG expression for each patient was calculated as the individual unweighted average difference in Ct (ΔCt) between the RGs (only those that were detected in that subject) and the three housekeeping genes.

### Statistical Analysis

A comprehensive descriptive analysis was performed by reporting mean ± standard deviation for continuous variables, and absolute and percentage numbers for categorical variables. The relapse rate was calculated as the number of events per 100 person-years, and the median disease-free time was assessed using the Kaplan-Meier method. The association between the independent variables of interest (CSC frequency and RG expression) and disease-free survival was assessed using a multivariable Cox regression model with robust standard errors.^9,25^ The results were reported as hazard ratio (HR) associated with a 1% increase in CSC frequency or with a 1 Ct decrease in RG expression (which corresponds to an absolute increase in RG expression). Effect modification was assessed by adding interaction terms within the models’ equations. In the presence of effect modification, stratified HRs were calculated as linear combinations of model parameters. Effect modification of CSC frequency by clinical stage was a priori assumed to be present, based on the results shown in our recent research.^9^ Both unadjusted and confounder-adjusted HRs were reported, considering sex (male vs female), clinical stage (early vs locally advanced), and tumor histotype (ACL vs SCCL) as potential confounding variables. Statistical analyses were carried out using R 3.6.3 software (the R Foundation for Statistical Computing, Wien, Austria) at the 95% confidence level (*P* < .05).

## Results

Characteristics of patients and surgical specimens are reported in Table 1. The average age was 70.0 ± 9.3 years, 63.6% of patients were male, and all were smokers. There were 12 (54.5%) patients with early-stage NSCLC and 10 (45.5%) with locally advanced NSCLC, and the ACL histotype was more frequent (77.3%) than SCCL (22.7%). The average CSC frequency was equal to 4.0% ± 3.4%. The average expression of RG in CSC was 5.4 ± 2.4 ΔCt, whereas the average expression of RG in cancer cells was lower and was equal to 6.4 ± 3.7 ΔCt. The total follow-up time was equal to 23.7 person-years, with an average follow-up time equal to 394 days (range: 19-902 days). During this period, 17 patients (77.3%) experienced recurrence, with an incidence rate equal to 71.6 events per 100 person-years and with a median disease-free survival time equal to 0.92 years.

**Table 1.** Characteristics of patients.

	All patients (*n* = 22)	Early-stage patients (*n* = 12)	Locally advanced-stage patients (*n* = 10)
Characteristics of patients			
Age, years			
Mean ± SD	70.0 ± 9.3	71.9 ± 9.6	67.6 ± 8.9
Median (IQR)	70 (63-75)	71 (65-82)	70 (61-74)
Gender, male, *n* (%)	14 (63.6%)	6 (50.0%)	8 (80.0%)
Smoking habit (current or former), *n* (%)	22 (100.0%)	12 (100.0%)	10 (100.0%)
Characteristics of tumors			
Pathological stage, *n* (%)			
Stage I	7 (31.8%)	7 (58.3%)	-
Stage II	5 (22.7%)	5 (41.7%)	-
Stage IIIA	10 (45.5%)	-	10 (100.0%)
T, *n* (%)			
T1	6 (27.3%)	6 (50.0%)	-
T2	6 (27.3%)	6 (50.0%)	-
T3	3 (13.6%)	-	3 (30.0%)
T4	7 (31.8%)	-	7 (70.0%)
N, *n* (%)			
N0	19 (86.4%)	11 (91.7%)	8 (80.0%)
N1	2 (9.1%)	1 (8.3%)	1 (10.0%)
N2	1 (4.5%)	0 (0.0%)	1 (10.0%)
M0, *n* (%)	22 (100.0%)	12 (100.0%)	10 (100.0%)
Histotype, *n* (%)			
ACL	17 (77.3%)	8 (66.7%)	9 (90.0%)
Acinar	9 (40.2%)	5 (41.7%)	4 (40.0%)
Papillary	3 (13.6%)	1 (8.3%)	2 (20.0%)
Solid	5 (22.7%)	2 (16.7%)	3 (30.0%)
SCCL	5 (22.7%)	4 (33.3%)	1 (10.0%)
Pleural invasion (Yes), *n* (%)	11 (50.0%)	3 (25.0%)	8 (80.0%)
Vascular invasion (Yes), *n* (%)	3 (13.6%)	0 (0.0%)	3 (30.0%)
PET SUV_max_			
Mean ± SD	8.4 ± 5.9	5.7 ± 3.8	11.7 ± 6.4
Median (IQR)	5.8 (4.2-12.4)	5.3 (3.8-6.2)	11.5 (5.9-16.2)
Tumor dimension, mm			
Mean ± SD	51.6 ± 23.4	34.8 ± 16.1	71.7 ± 11.9
Median (IQR)	51 (29-70)	31 (25-40)	69 (63-80)
Characteristics of surgery			
Type of surgery, *n* (%)			
Lobectomy	19 (86.4%)	12 (100.0%)	7 (70.0%)
Pneumonectomy	3 (13.6%)	2 (16.7%)	3 (30.0%)
Surgical approach, *n* (%)			
Lateral thoracotomy	15 (68.2%)	5 (41.7%)	10 (100.0%)
VATS	7 (31.8%)	7 (58.3%)	0 (0.0%)
Diagnostic procedures, *n* (%)			
^18^F-FDG PET/CT + FBS	11 (50.0%)	11 (91.7%)	0 (0.0%)
^18^F-FDG PET/CT + FBS + EBUS	11 (50.0%)	1 (8.3%)	10 (100.0%)
Previous treatments, *n* (%)			
Neoadjuvant CT	3 (13.6%)	0 (0.0%)	3 (30.0%)
Neoadjuvant RT	0 (0.0%)	0 (0.0%)	0 (0.0%)
Adjuvant CT	11 (50.0%)	2 (16.7%)	9 (90.0%)
Adjuvant RT	0 (0.0%)	0 (0.0%)	0 (0.0%)
Cellular and molecular characteristics			
CSCs frequency, % on viable cells			
Mean ± SD	4.0 ± 3.4%	5.1% ± 3.3%	3.4% ± 3.6%
Median (IQR)	3.4% (1.3-4.7%)	3.7% (2.6-6.8%)	2.4% (0.9-4.2%)
Expression of RGs in CSCs ∆Ct			
Mean ± SD	5.4 ± 2.4	5.5 ± 2.5	5.4 ± 2.5
Median (IQR)	6.0 (3.5-7.3)	6.0 (3.6-7.5)	5.9 (3.6-7.1)
Expression of RGs in cancer cells ∆Ct			
Mean ± SD	6.4 ± 3.7	6.9 ± 4.0	5.7 ± 3.5
Median (IQR)	6.1 (3.9-8.4)	6.5 (4.8-9.5)	6.1 (3.4-7.3)

Abbreviations: ∆Ct, individual unweighted average difference in Ct between RGs and housekeeping genes; ^18^F-FDG PET/CT, positron emission tomography with 2-deoxy-2-[fluorine-18]fluoro-d-glucose integrated with computed tomography; ACL, adenocarcinoma of the lung; CSCs, cancer stem cells; CT, chemotherapy; Ct, cycle threshold; EBUS, endobronchial ultrasound; FBS, fibrobronchoscopy; IQR, interquartile range; RG, recurrence-related gene; RT, radiotherapy; SCCL, squamous cell carcinoma of the lung; SD, standard deviation; VATS, video-assisted thoracic surgery.

### Influence of CSC Frequency and RG Expression on Disease-free Survival

First, we assessed the effect modification of CSC frequency and RG expression related to clinical stage and tumor histotype. Based on the statistical significance of interaction terms and on previous results,^9^ we assumed the presence of effect modification of CSC frequency by clinical stage (*P* = .139 in the present study). Conversely, the effect of RG expression was not modeled as different among clinical stages and histotypes, whether RG expression was measured in CSC (*P* = .978 and *P* = .367, respectively) or in cancer cells (*P* = .865 and *P* = .218, respectively), and the effect of CSC frequency was similar between histotypes (*P* = .976). The adjusted analysis gave the following results: CSC frequency was not related to disease-free survival in early-stage patients (HR = 0.84, 95%CI = 0.53-1.33, *P* = .454), whereas it was a risk factor for locally advanced-stage patients (HR = 1.22, 95%CI = 1.09-1.35, *P* = .000); and RG expression—if measured in CSC—was related to a higher risk of recurrence (HR = 1.19, 95%CI = 1.03-1.39, *P* = .021) (Table 2). In this analysis, CSC frequency and RG expression were two independent predictors of disease-free survival, as their interaction terms were not statistically significant in either early- or locally advanced-stage patients (*P* = .548 and *P* = .858, respectively). Finally, the effect of RG expression in cancer cells on disease-free survival was lower and was not statistically significant (HR = 1.12, 95%CI = 0.94-1.33, *P* = .196), while the effect of CSC frequency in locally advanced-stage patients was confirmed (HR = 1.25, 95%CI = 1.09-1.44, *P* = .001) (Table 2).

**Table 2.** Effect of CSC frequency and expression of recurrence genes score on recurrence-free survival.

	Unadjusted model^a^			Adjusted model^b^		
	HR	95% CI	*P*-value	HR	95% CI	*P*-value
Expression of RG measured in CSC						
Effect of +1% CSC frequency						
in early-stage patients	0.99	0.78-1.24	.911	0.84	0.53-1.33	.454
in locally advanced-stage patients	1.23	1.07-1.41	.003^*^	1.22	1.09-1.35	.000^*^
Effect of −1 Ct in RG expression	1.16	0.99-1.36	.076	1.19	1.03-1.39	.021^*^
Expression of RG measured in cancer cells						
Effect of +1% CSC frequency						
in early-stage patients	1.03	0.80-1.32	.839	0.91	0.54-1.53	.728
in locally advanced-stage patients	1.27	1.10-1.48	.001^*^	1.25	1.09-1.44	.001^*^
Effect of −1 Ct in RG expression	1.12	0.99-1.26	.065	1.12	0.94-1.33	.196

The table reports the average effect of a 1% increase in CSC frequency and of an increase in recurrence genes expression (1 Ct decrease) on disease-free survival.

The unadjusted model has the following independent variables: CSC frequency (%), stage (early, locally advanced), CSC frequency × stage interaction, RG expression (Ct); ^b^The adjusted model is equal to the base model but it further adjusts for gender (male, female) and histotype (adenocarcinoma, squamous cell carcinoma).

Statistically significant at 95% confidence level (*P* < .05).

Abbreviations: CI, confidence interval; CSC, cancer stem cells; Ct, cycle thresholds; HR, hazard ratio.

## Discussion

Recurrence is still a persistent problem and a point of discussion all over the world for every type of cancer.^9,26-28^ In particular, with regard to molecular biology, the scientific community is currently focused on CSCs, which seem to have a strategic role in tumor growth, progression, and relapse.^27,29^ These subpopulations have been identified^5,30,31^ and studied for their ability to grow under stringent conditions in vitro, as an example of their highest expression of aggressiveness.^5^ However, the connection between these cells and cancer recurrence is still uncertain.^32^ The importance to target CSCs has been strongly supported by our recent prospective observational study^9^ which analyzed the role of the frequency of ALDH^high^ cells (CSCs) in surgical patients who developed recurrence.^9^ In that research, we found that a 1% increase in the frequency of CSCs in locally advanced patients yielded a 26% increase in the hazard of relapse, indicating that CSC frequency could represent a strong predictor variable for patient prognosis.^9^

In the last decades, researchers have also started to think of cancer as a “genetic condition” derived from the mutation or alteration of multiple genes expressed in solid tumors.^33,34^ This consideration has led to the investigation and discovery of several genes related to cancer development and recurrence.^35,36^ In particular, in 2015, Bueno et al^21^ defined a prognostic score based on cell cycle genes related to recurrence. This score was shown to be able to stratify the risk of death in patients undergoing surgery for early stages of ACL.^21^ Several studies were also carried out on solid tumors to define new diagnostic and therapeutic options based on the stratification of gene expression, although there is currently no globally accepted molecular score for predicting disease-free survival in different types of cancer.^37-39,40^

We recently carried out a cross-sectional study to measure the expression of cell cycle genes identified by Bueno et al^21^ in CSCs isolated from patients undergoing surgery for early and locally advanced stages of ACL and SCCL. In that study, an overexpression of these genes in CSC compared to cancer cells was observed, particularly in early stages of ACL and SCCL.

Based on those studies,^4,5,9,21,22,29,30^ we further analyzed data from our previous prospective cohort study, to assess the joint prognostic role of CSC frequency and RG expression on disease-free survival.

Firstly, the CSC frequency was confirmed to be a risk factor for locally advanced patients, as a 1% increase in the frequency of CSCs yielded a 22% increase in the hazard of relapse. This result was expected, as the present study analyses, a subset of patients enrolled in our previous prospective cohort study.^9^ Secondly, the RG expression was assessed as a risk factor for disease-free survival, in two different ways. In the first one, RG expression was measured in CSC, whereas in the second one the RG expression was measured in cancer cells. We observed that RG expression measured in CSC was a risk factor itself, as a 1 Ct decrease yielded on average a 19% increase in the hazard of relapse. Conversely, the RG expression measured in cancer cells was not significantly associated with disease-free survival, highlighting a poorer prognostic value of RG expression measured in cancer cells. These findings may suggest that the cell cycle genes proposed by Bueno et al^21^ have a prognostic value not only for early stages of ACL but also for locally advanced stages of ACL and for SCCL, although further large-scale studies are needed to confirm this hypothesis.

Moreover, CSC and RG were shown to be independent risk factors, suggesting that they need to be considered jointly for disease-free survival prediction and stratification, especially in locally advanced stages of ACL and SCCL. Considering these findings, we believe that the presence of these cell cycle genes in CSCs, and to a much greater extent the frequency of CSC may be considered for the design and development of a prognostic score calibrated on cancer relapse. Moreover, our data may generate hypotheses for the development of targeted therapies against CSCs, in order to improve the major pathological responses to standard treatments. Notably, the possibility of targeting early and locally advanced stages of NSCLC may allow the reduction of tumor growth, with a better control of tumor development. In addition, our study may also contribute to the knowledge about the metastatization process.

### Limitations

The results of this study are limited mainly by the low number of included patients and by the high heterogeneity in their characteristics. The inclusion of patients with cancer stages ranging from I to IIIA, as well as that of both ACL and SCCL histotypes may indeed have affected the generalizability of our findings, even if the effect modification of such variables on the risk of relapse was duly assessed and reported. Moreover, there is uncertainty on the role of CSC and RG on disease-free survival in non-smokers, as in our prospective cohort study all enrolled patients happened to be current or former smokers. Finally, selection bias and confounding bias cannot be definitely ruled out in an observational study. Further large-scale studies are needed to better understand the prognostic role of CSC and RG in lung cancer.
